# High‐contiguity genome assembly of the chemosynthetic gammaproteobacterial endosymbiont of the cold seep tubeworm *Lamellibrachia barhami*

**DOI:** 10.1111/1755-0998.13220

**Published:** 2020-07-24

**Authors:** Corinna Breusing, Darrin T. Schultz, Sebastian Sudek, Alexandra Z. Worden, Curtis Robert Young

**Affiliations:** ^1^ Monterey Bay Aquarium Research Institute Moss Landing CA USA; ^2^ National Oceanography Centre Southampton UK; ^3^ Department of Biomolecular Engineering and Bioinformatics University of California Santa Cruz Santa Cruz CA USA; ^4^ GEOMAR Helmholtz Centre for Ocean Research Kiel Germany

**Keywords:** chemosynthetic symbiont, Hi‐C, high‐contiguity genome assembly, *Lamellibrachia barhami*, long‐read sequencing

## Abstract

Symbiotic relationships between vestimentiferan tubeworms and chemosynthetic Gammaproteobacteria build the foundations of many hydrothermal vent and hydrocarbon seep ecosystems in the deep sea. The association between the vent tubeworm *Riftia pachyptila* and its endosymbiont *Candidatus* Endoriftia persephone has become a model system for symbiosis research in deep‐sea vestimentiferans, while markedly fewer studies have investigated symbiotic relationships in other tubeworm species, especially at cold seeps. Here we sequenced the endosymbiont genome of the tubeworm *Lamellibrachia barhami* from a cold seep in the Gulf of California, using short‐ and long‐read sequencing technologies in combination with Hi‐C and Dovetail Chicago libraries. Our final assembly had a size of ~4.17 MB, a GC content of 54.54%, 137X coverage, 4153 coding sequences, and a checkm completeness score of 97.19%. A single scaffold contained 99.51% of the genome. Comparative genomic analyses indicated that the *L. barhami* symbiont shares a set of core genes and many metabolic pathways with other vestimentiferan symbionts, while containing 433 unique gene clusters that comprised a variety of transposases, defence‐related genes and a lineage‐specific CRISPR/Cas3 system. This assembly represents the most contiguous tubeworm symbiont genome resource to date and will be particularly valuable for future comparative genomic studies investigating structural genome evolution, physiological adaptations and host‐symbiont communication in chemosynthetic animal‐microbe symbioses.

## 1 INTRODUCTION

Mutualistic symbioses between chemoautotrophic bacteria and invertebrate animals sustain deep‐sea hydrothermal vent and cold seep ecosystems worldwide (Dubilier, Bergin, & Lott, 2008). Among the key fauna are vestimentiferan tubeworms (Polychaeta; Siboglinidae), which act as foundation species by creating biomass‐rich aggregations that provide habitat space and ecological niches for a variety of other co‐occurring animal taxa (Bright & Lallier, 2010). Adult tubeworms do not possess a functional digestive tract and are nutritionally dependent on their gammaproteobacterial endosymbionts that are housed in a specialized organ within the coelomic cavity (trophosome). Through the oxidation of sulphide or hydrogen these symbionts gain chemical energy to convert inorganic carbon to organic matter, which serves as food for the host (Petersen et al., 2011; Thiel et al., 2012).

The ecophysiology, environmental transmission mode, population structure and genomics of the vent‐dwelling symbiont *Candidatus* Endoriftia persephone, especially in association with its host *Riftia pachyptila*, have been investigated extensively (e.g., Nussbaumer, Fisher, & Bright, 2006; Markert et al., 2007; Robidart et al., 2008; Robidart, Roque, Song, & Girguis, 2011; Gardebrecht et al., 2012; Klose et al., 2015; Perez & Juniper, 2016; Hinzke et al., 2019). By contrast, comparatively little is known about the biology of symbionts associated with other tubeworm species, in particular those that are usually found at cold seeps. Aspects of the evolution, physiology and ecology of these symbionts can be expected to be different from that of vent‐associated symbionts, given that seeps are sedimented habitats that differ in physicochemical conditions and environmental stability from hydrothermal vents (Bright & Lallier, 2010). In addition, seep‐associated host species exhibit slower growth rates and longer lifespans than their vent relatives and develop extensive subsurface root systems that play important roles in the energy cycles of both host and symbiont (Cordes, Arthur, Shea, Arvidson, & Fisher, 2005; Boetius, 2005).

To date, four seep tubeworm symbiont genomes from the Gulf of Mexico and the South China Sea (*Escarpia spicata*, *Lamellibrachia luymesi*, *Seepiophila jonesi*, *Paraescarpia echinospica* symbionts) and three vent tubeworm symbiont genomes from the East Pacific Rise and the Juan de Fuca Ridge (*Riftia pachyptila*, *Ridgeia piscesae*, *Tevnia jerichonana* symbionts) have been published (Gardebrecht et al., 2012; Perez & Juniper, 2016; Li, Liles, & Halanych, 2018; Yang et al., 2019). Recent comparative genomic analyses based on these genomes suggest that seep‐associated tubeworm symbionts use the same carbon fixation and sulphur oxidation pathways as vent‐associated tubeworm symbionts, but might have a higher potential to acquire foreign genetic material, contain a larger amount of virulence factors for modulating host‐symbiont interactions and utilize a more diverse repertoire of energy sources for their metabolism (Li et al., 2018; Yang et al., 2019). However, since seep tubeworms have broad geographic distributions and can occur at other types of chemosynthetic habitats (e.g., McMullin, Hourdez, Schaeffer, & Fisher, 2003; Reveillaud, Anderson, Reves‐Sohn, Cavanaugh, & Huber, 2018), genomic analyses on their symbionts from a variety of biogeographic regions are needed to better understand the links between symbiont metabolic capacities, diversity, evolution and host niche utilization. In addition, due to the difficulties associated with metagenomic data analysis, the currently available symbiont genome assemblies have varying degrees of fragmentation, which complicates comparative genomic investigations of structural rearrangements, such as gene duplications, translocations or inversions.

**FIGURE 1:**
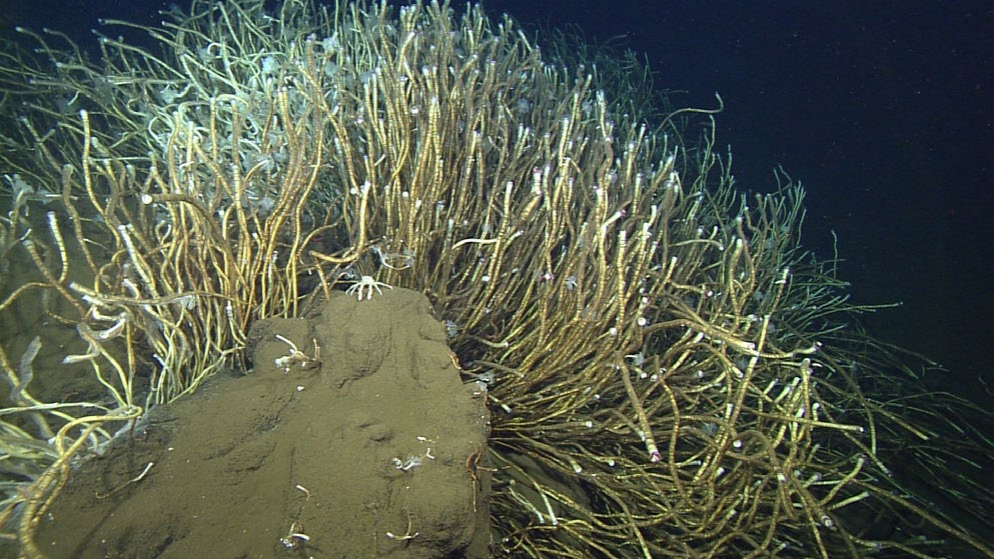
*Lamellibrachia barhami* tubeworms at a cold seep in the Pescadero Transform Fault (Gulf of California). The image is provided with courtesy of Bob Vrijenhoek and the Monterey Bay Aquarium Research Institute

To address these limitations and improve assembly contiguity, we sequenced and scaffolded the symbiont genome of the tubeworm species *Lamellibrachia barhami* (Figure 1) from a hydrocarbon seep that was recently discovered at the Pescadero Transform Fault in the southern Gulf of California (Goffredi et al., 2017; Paduan et al., 2018; Clague et al., 2018). Our approach combined Illumina shotgun, Nanopore and Hi‐C/Chicago data to generate a chromosome‐level assembly, which we compared against previously published tubeworm endosymbiont genomes (Table 1).

**TABLE 1:** General information about vestimentiferan symbiont genomes compared in this study

Symbiont of:	Accession No.	Genome size (Mb)	No. of contigs	N50 (Mb)	Habitat	Reference
*Lamellibrachia barhami*	JAAVSH000000000	4.17	19	4.15	Seep	This study
*Escarpia spicata*	QFXE00000000	4.06	23	0.31	Seep	Li et al. (2018)
*Lamellibrachia luymesi*	QFXD00000000	3.53	337	0.02	Seep	Li et al. (2018)
*Paraescarpia echinospica*	RZUD00000000	4.06	14	0.38	Seep	Yang et al. (2019)
*Seepiophila jonesi*	QFXF00000000	3.53	323	0.02	Seep	Li et al. (2018)
*Ridgeia piscesae*	LDXT00000000	3.44	97	0.08	Vent	Perez and Juniper (2016)
*Riftia pachyptila*	AFOC00000000	3.48	197	0.03	Vent	Gardebrecht et al. (2012)
*Tevnia jerichonana*	AFZB00000000	3.64	184	0.10	Vent	Gardebrecht et al. (2012)

## 2 MATERIALS AND METHODS

### 2.1 Sample collection and DNA methods

Tubeworm specimens were collected from three eastern Pacific seep sites with the remotely operated vehicles (ROV) *Doc Ricketts* and *Tiburon* during the R/V *Western Flyer* 2002, 2012 and 2015 cruises (Table 2). Sampling permits for expeditions in US territorial waters were not needed, while permits for collections in the Gulf of California were obtained by the Monterey Bay Aquarium Research Institute from Mexico's Secretariat of Foreign Affairs (SRE: CTC‐00130, CTC/01700/15), the Secretariat of Agriculture and Rural Development and the National Commission of Fisheries and Aquaculture (SAGARPA/CONAPESCA: DGOPA‐DAPA/2/818/010212/140, DGOPA‐02919/14). Upon recovery of the ROVs, tubeworms were quickly excised from their tubes, dissected and frozen at –80°C. We considered only individuals with intact trophosomes for further analysis. Genomic DNA was extracted from symbiont‐bearing trophosome tissues using the QIAGEN DNeasy Blood & Tissue kit (Qiagen, Hilden, Germany) and further purified with the PowerClean Pro DNA clean‐up kit (Mo Bio, Carlsbad, CA, USA). To obtain high‐molecular weight (HMW) DNA we also performed extractions using a CHAOS buffer protocol (Supporting Information). The tubeworm host species were identified based on their mitochondrial cytochrome‐c‐oxidase I (*COI*) sequences, which we amplified and sequenced with the primer pairs jgLCO1490 and jgHCO2198 (Geller, Meyer, Parker, & Hawk, 2013) following previously published PCR protocols (Breusing, Johnson, Tunnicliffe, & Vrijenhoek, 2015). Sequences were edited in geneious v9.1.8 (http://www.geneious.com/) and annotated via blast v2.9.0+ searches (Camacho et al., 2009).

**TABLE 2:** Sampling information for *Lamellibrachia barhami* in the eastern Pacific Ocean

Locality	Latitude	Longitude	Depth (m)	Dive #^a^	N^b^	Year
Mendocino	40°21'N	125°13'W	1,578	T: 448	1	2002
Pinky’s Vent	27°35'N	111°29'W	1,572	D: 380	2	2012
Pescadero Transform Fault	23°38'N	108°23'W	2,381–2,390	D: 756	4	2015

^a^ Submersibles: D, *Doc Ricketts*; T, *Tiburon*;

^b^ N, sample size.

### 2.2 Illumina high‐throughput sequencing and read preparation

Barcoded 2 × 125 bp paired‐end metagenomic libraries of three *L. barhami* individuals from the Pescadero Transform Fault were prepared and sequenced on ~12% of an Illumina HiSeq2500 lane at the Huntsman Cancer Institute at the University of Utah. Sequences were investigated for quality with fastqc v0.11.5 (Andrews, 2010) and then adapter‐clipped and quality‐trimmed with Trimmomatic v0.36 (Bolger et al., 2014) using a custom adapter file and the following options: slidingwindow:4:20 leading:5 trailing:5 minlen:50. PhiX and human contaminating reads were removed using bowtie2 v2.3 (Langmead & Salzberg, 2012). Mapping analysis to preliminary genome assemblies (described below) of the *L. barhami* endosymbiont indicated that individual D756‐A12‐LB11 yielded an exceptionally high amount of symbiont reads (~40% as opposed to ~1% in other specimens) and we therefore targeted this specimen for further genomic analyses (Table 3).

**TABLE 3:** Summary of individuals and sequencing reads used for the genome assembly

Sample	Illumina reads				Nanopore reads				Application
	Raw	Filtered	Symbiont	% Sym	Raw	Filtered	Symbiont	% Sym	
D756‐A12‐LB11	13031048	10334074	3999526	38.70	944577	941994	178703	18.97	Genome assembly
D756‐A12‐LB10	12423660	9727530	129486	1.33	–	–	–	–	Differential coverage binning
D756‐LB3	2189648	1785842	23212	1.30	–	–	–	–	Differential coverage binning
D756‐LB7	–	–	–	–	1049405	1044613	121927	11.67	Gap filling
T448‐A2‐3		74647466	5908892	7.92	–	–	–	–	Two Chicago libraries (*DpnII* + *FatI*), scaffolding
D380‐A2‐15A		45017644	3568662	7.93	–	–	–	–	One Hi‐C library (*FatI*), scaffolding
D380‐A2‐14G		30785152	3500416	11.37	–	–	–	–	One Hi‐C library (*DpnII*), scaffolding

### 2.3 Nanopore long‐read sequencing and read preparation

To assist the resolution of repeat regions in the symbiont genome, we sequenced ~1 million long reads of D756‐A12‐LB11 using Oxford Nanopore Technologies (ONT) sequencing. Due to tissue and HMW DNA limitations for this specimen, we sequenced an additional ~1 million reads of a second *L. barhami* individual from the Pescadero Transform Fault (Table 3) that contained the same *16S* rRNA symbiont phylotype (C. Breusing, unpublished data). ONT sequencing was performed on a MinION device following library preparation with the SQK‐LSK108 and SQK‐RAD003 sequencing kits (Oxford Nanopore, Oxford, UK). Reads were locally base‐called and converted to FASTQ format with albacore v2.3.4 (Oxford Nanopore, Oxford, UK). Adapters were removed with porechop v0.2.4 (https://github.com/rrwick/Porechop).

### 2.4 Hybrid metagenome assembly and binning

We used idba‐ud v1.1.3 (Peng, Leung, Yiu, & Chin, 2012) to initially create a combined draft metagenomic assembly from all sequenced individuals, choosing k‐mers between 21 and 121 at a step size of 10 and a minimum support of 2. Reads were corrected before assembly with the ‐‐pre_correction option. Binning of the assembly was performed with gbtools v2.6.0 (Seah & Gruber‐Vodicka, 2015) based on differential coverage following guidelines by the authors (https://github.com/kbseah/genome-bin-tools/wiki). To improve the quality of the genome, we separated all symbiont‐related sequences from the metagenomic data set by mapping the Illumina and Nanopore sequences against the draft assembly with BBMap v38.23 (https://sourceforge.net/projects/bbmap/) and Minimap2 v2.17 ( li, 2018), respectively. We then performed a second assembly using only the mapped symbiont reads of individual D756‐A12‐LB11 in spades v3.13.1 (Bankevich et al., 2012) based on k‐mer sizes between 21 and 111 at an increment of 10. Binning was done as described above.

### 2.5 Genome scaffolding and annotation

Our assembly approach resulted in 328 prescaffolds. To increase the contiguity of the genome, we concentrated GC‐rich symbiont DNA via CsCl‐bisbenzimide gradient centrifugation (Tran‐Nguyen & Schneider, 2013; Supporting Information ), and then prepared four Hi‐C and two Chicago libraries for three *L. barhami* individuals using the *FatI* and *DpnII* restriction enzymes (Belton et al., 2012; Putnam et al., 2016). After assessing library performance on a MiSeq system, four successful libraries were sequenced on an Illumina HiSeq4000 system with a 2 × 150 bp paired‐end protocol (Table 3).

The juicer v1.5 and 3d‐dna v180922 pipelines (Durand et al., 2016; Dudchenko et al., 2017) were subsequently applied for scaffolding using long‐range linkage information provided by the Hi‐C and Chicago data.

To reduce assembly errors, we re‐assembled and ‐scaffolded the genome for D756‐A12‐LB11 as described above, using only reads that mapped to the Hi‐C/Chicago scaffolds. The final assembly was re‐binned and polished with pilon v1.22 (Walker et al., 2014). Symbiont Nanopore reads from the second *L. barhami* individual were used for gap‐filling with lr_gapcloser (Xu et al., 2019). Gene prediction and functional annotation was performed with rast v2.0 (Aziz et al., 2008; Overbeek et al., 2014; Brettin et al., 2015). To detect metabolic enzymes that were missing in the rast annotations and infer functions of hypothetical proteins, we further compared all predicted gene sequences against the Swiss‐Prot and RefSeq databases using blastp v2.9.0+ (Camacho et al., 2009). Detected hydrogenases were classified with hyddb (Søndergaard, Pedersen, & Greening, 2016). Assembly quality and completeness were assessed with quast v5.0.0 (Gurevich, Saveliev, Vyahhi, & Tesler, 2013) and checkm v1.0.18 (Parks, Imelfort, Skennerton, Hugenholtz, & Tyson, 2015) based on 280 Gammaproteobacteria‐specific single copy marker genes. To determine potential genetic heterogeneity in the endosymbiont population, we identified single nucleotide variants (SNVs) with angsd v0.920 (Korneliussen, Albrechtsen, & Nielsen, 2014) after mapping the symbiont Illumina reads of D756_A12_LB11 to the final assembly. To remove spurious SNVs from the analysis we used only reads that mapped in proper pairs, resulted in unique alignments, achieved minimum PHRED‐scaled mapping qualities of 30 after adjustment for excessive mismatches and had minimum PHRED‐scaled base qualities of 20. Q scores were adjusted in indel regions by calculating per‐base alignment qualities. We further considered only sites with read depths between 20 and 200 (based on the global read depth distribution) and with *p*‐values of 1e‐6.

### 2.6 Comparative genomics and phylogenomics

A phylogeny of the symbiont *16S* rRNA gene sequences was constructed with mrbayes v3.2.7a (Huelsenbeck & Ronquist, 2001) via the Cipres Science Gateway v3.3 (Miller, Pfeiffer, & Schwartz, 2010) implementing the GTR + I + G substitution model (Thornhill et al., 2008). Input NEXUS files were prepared by aligning the symbiont *16S* rRNA sequences against the global Silva *16S* rRNA alignment with Sina v1.2.11 (Pruesse, Peplies, & Glöckner, 2012). Two runs of four chains (three heated plus one cold) were run for 1,100,000 generations at a sampling interval of 100 generations and a burnin of 100,000 generations. The symbiont *16S* rRNA gene sequence of the frenulate *Galathealinum brachiosum* was used as outgroup (Li et al., 2018). MCMC convergence was assessed with tracer v1.7.1 (Rambaut, Drummond, Xie, Baele, & Suchard, 2018) and the consensus tree was displayed with figtree v1.4.4 (http://tree.bio.ed.ac.uk/software/figtree/). To infer evolutionary associations on a genomic level and to identify unique and core genomic features of the *L. barhami* symbiont, we followed the anvi’o v6.2 pangenomics workflow (Eren et al., 2015; Delmont & Eren, 2018), using external gene calls obtained with rast for all symbiont genomes. We used the “‐‐ncbi‐blast” option to compute amino acid sequence similarities and the MCL algorithm (van Dongen & Abreu‐Goodger, 2012) for clustering with the following settings: minbit = 0.5, mcl‐inflation = 2, min‐occurrence = 1. progressivemauve v20150213 (Darling, Mau, & Perna, 2010) was used to assess structural rearrangements between symbiont genomes based on an empirically determined LCB weight of 30,000. The *L. barhami* symbiont genome was chosen as reference for contig reordering of the other symbiont assemblies before structural analysis.

Summary graphics for statistical analyses were produced in r v3.5.2 (R Core Team, 2018) using the ggplot2, plotly and hh packages (Wickham, 2016; Heiberger, 2020; Sievert, 2020). All images were polished in INKSCAPE v1.0 (https://inkscape.org).

## 3 RESULTS

### 3.1 Genome assembly

Our sequencing approach in the candidate individual D756‐A12‐LB11 yielded ~3.9 million symbiont Illumina sequences and ~180,000 symbiont Nanopore sequences for assembly, which were joined into 19 scaffolds with the help of ~13 million Hi‐C and Chicago reads (Table 3 and 4). The final assembly had a size of ~4.17 Mb, with a GC content of 54.54%, an average coverage of 137X, a scaffold N50 of ~4.15 Mb and an L50 of 1 (Table 4). A single scaffold of ~4.15 Mb comprised the majority of the genome (99.51%), indicating high contiguity of the assembly (Table 4). Assessment of genome completeness based on 280 Gammaproteobacteria‐specific single copy marker genes resulted in a completeness score of 97.19% and a low level of contamination (3.55%) that appeared to be caused by the presence of strain variation (Table 4; Parks et al., 2015). Our variant analyses indicated a total of 28,505 polymorphic sites in the genome, resulting in an average density of 6.84 SNVs per kbp.

**TABLE 4:** Assembly statistics for the *Lamellibrachia barhami* endosymbiont genome

Assembly metric	
Number of chromosomes	1
Genome size (bp)	4,169,104
Percent assembled	99.51
Number of scaffolds	19
Longest scaffold (bp)	4,148,554
Scaffold N50	4,148,554
Scaffold L50	1
Number of contigs	197
Contig N50	37,262
Contig L50	34
GC (%)	54.54
Ns per 100 kbp	1101.92
Average coverage (X)	137
Illumina coverage (X)	111
Nanopore coverage (X)	26
Number of coding sequences	4,153
Number of RNAs	47
Completeness (%)	97.19
Contamination (%)	3.55
Strain heterogeneity (%)	66.67

### 3.2 Genome annotation

**FIGURE 2:**
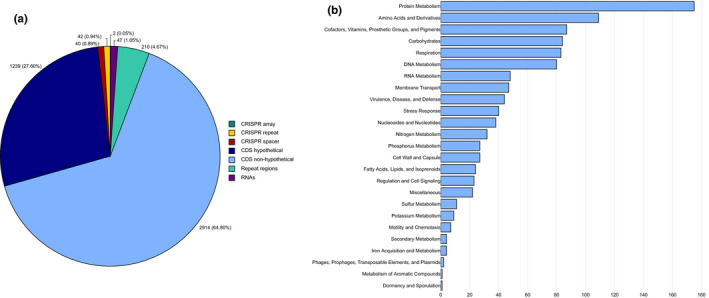
(a) General overview of genome features in the *Lamellibrachia barhami* endosymbiont. (b) Classification of nonhypothetical CDS into subsystems. Some features belonged to more than one category

The *L. barhami* symbiont genome comprises 4,153 predicted genes, 47 RNAs, 210 repeat regions and two CRISPR arrays comprising a total of 42 repeats and 40 spacers (Figure 2a). 2,914 of the predicted genes were functionally annotated, while the remaining 1,239 genes were classified as hypothetical/uncharacterized proteins (Table S1; Figure 2b). 898 individual genomic features (coding sequences and RNAs) were categorized into 25 broader subsystems, in particular protein, amino acid, cofactor, DNA, and carbon metabolism as well as cellular respiration (Table S2; Figure 2b).

### 3.3 Comparative genomics and phylogenomics

**FIGURE 3:**
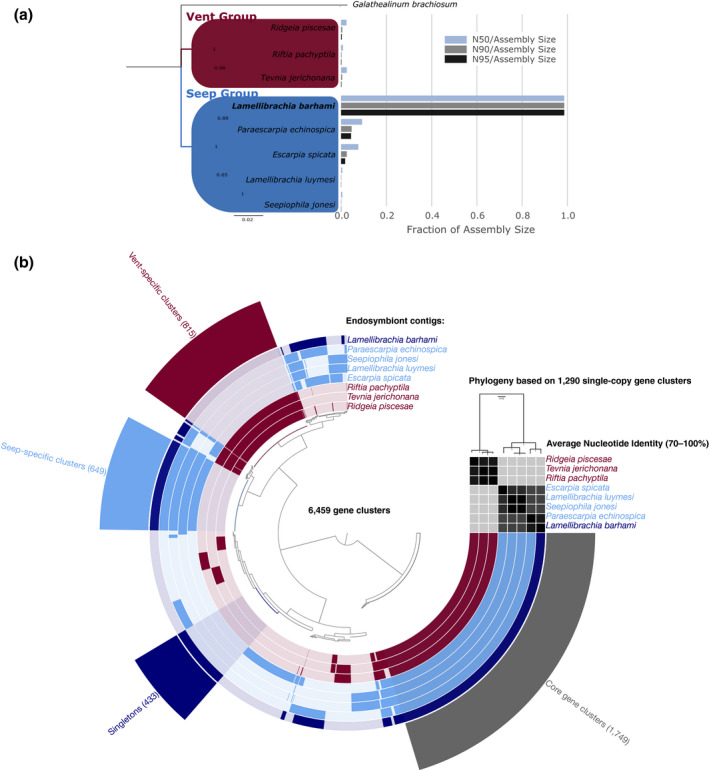
(a) Bayesian phylogeny of the *16S* rRNA gene for recently sequenced vestimentiferan endosymbionts. The *Lamellibrachia barhami* symbiont is closely related to the *Paraescarpia echinospica* symbiont within the Seep Group of vestimentiferan endosymbionts. The scale bar shows expected number of substitutions per site. The right side shows a comparison of assembly contiguity based on N50/N90/N95 metrics relative to assembly size. (b) Pangenomic comparison of available vent and seep symbiont genomes based on 6,459 gene clusters. The inner dendrogram shows hierarchical relationships among these clusters based on their distribution across genomes. Each circle layer represents a single symbiont genome, with the *L. barhami* symbiont shown in dark blue, other seep‐associated symbionts shown in light blue and vent‐associated symbionts shown in red. Within layers, dark colours indicate presence of a gene cluster, while light colours indicate absence. At the outside of the pangenome graph, different gene cluster groups and their abundances are highlighted, including core gene clusters among all symbiont genomes (grey), singleton gene clusters of the *L. barhami* symbiont (dark blue), vent‐specific gene clusters (dark red) and seep‐specific gene clusters (light blue). Midpoint‐rooted phylogenomic relationships among symbiont genomes are shown in the top right dendrogram based on 1,290 single copy gene clusters. The matrix below the dendrogram shows average nucleotide identities (70%–100%) among genomes in aligned regions, with light and dark grey colours indicating lower and higher similarities, respectively

**FIGURE 4:**
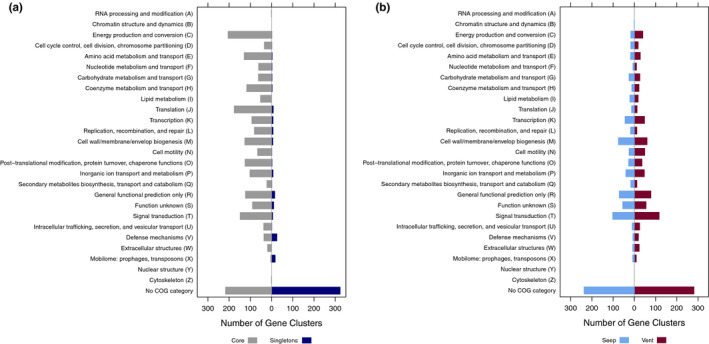
(a) Categorization of core gene clusters and singleton gene clusters of the *Lamellibrachia barhami* symbiont into Clusters of Orthologous Groups (COGs). (b) Functional categorization of vent‐ and seep‐specific gene clusters. Some gene clusters belonged to more than one category

Phylogenetic analyses of the *16S* rRNA gene and 1,290 orthologous single‐copy gene clusters indicated that the *L. barhami* symbiont belongs to the Seep Group of vestimentiferan endosymbionts and is closely related to the recently sequenced symbiont of the tubeworm species *Paraescarpia echinospica* from the South China Sea (Figures 3a,b). The genome of the *L. barhami* symbiont was characterized by a markedly higher contiguity than other assemblies and contained 433 unique gene clusters (consisting of 443 genes) (Figures 3a,b; Table S3). The majority of these gene clusters had unknown functions, while several others were involved in viral defence mechanisms and genetic transposition (Figure 4a; Table S3). Notably, we found different type I and type II restriction‐modification systems as well as a lineage‐specific CRISPR‐Cas3 system (type I‐E) associated with two distinctive CRISPR arrays, one consisting of 27 repeats and 26 spacers and another one consisting of 15 repeats and 14 spacers (Table S1, S2, S3). All genomes shared 1,749 core gene clusters (consisting of 15,149 genes), but differed in the presence or absence of 4,710 gene clusters (consisting of 14,792 genes) (Figure 3b; Table S4). The core genome is abundant in genes involved in energy production and conversion, translation, signal transduction, post‐translational modification, amino acid metabolism, cell wall biogenesis and a variety of genes without distinct functional classification (Figure 4a; Table S4). 649 gene clusters (consisting of 3,685 genes) were specific to seep tubeworm symbionts, whereas 815 gene clusters (consisting of 2,669 genes) were only found in vent‐associated symbionts (Figure 3b, 4b; Table S5, S6). These habitat‐specific clusters showed very similar distributions into functional categories for both vent and seep group. Although most of these gene clusters could not be classified, many were related to signal transduction, cell wall biosynthesis and transcription (Figure 4b).

**FIGURE 5:**
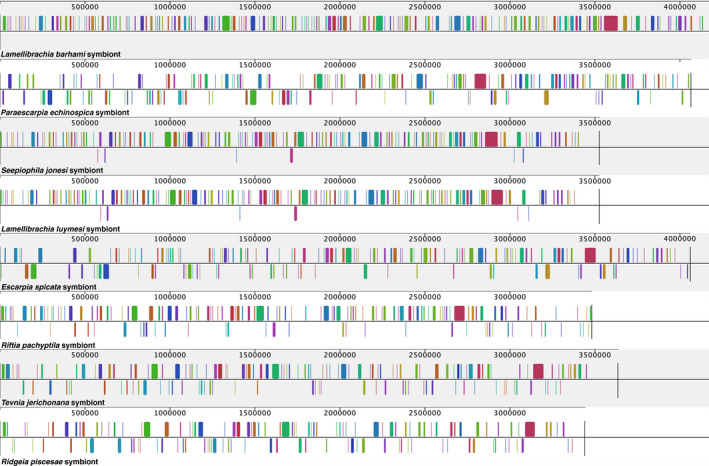
Structural rearrangements between tubeworm symbiont genomes. Coloured fragments indicate locally collinear blocks where sequences are homologous and show no structural variation. Fragments under the horizontal line indicate inversions. The *Lamellibrachia barhami* symbiont genome was used as alignment reference

In aligned regions, the average nucleotide identity (ANI) of the *L. barhami* symbiont genome was 91.73%–97.38% with other seep symbiont genomes and 75.35%–75.41% with vent symbiont genomes (Figure 3b; Table S7). Considering unaligned regions in the calculation, ANIs were markedly lower, 57.27%–81.87% with other seep symbiont genomes and only 22.40%–27.08% with vent symbiont genomes. Genome rearrangement analyses indicated that the genomes of all tubeworm symbionts are structurally highly distinct and are characterized by multiple translocations, inversions and indels (Figure 5). The only genomes that showed clear similarity in structure were those of the *Lamellibrachia luymesi* and *Seepiophila jonesi* symbionts.

### 3.4 Chemoautotrophic metabolism

Similar to other chemosynthetic Gammaproteobacteria, the *L. barhami* endosymbiont possesses key enzymes for sulphur oxidation via the Sox‐independent pathway (Nakagawa & Takai, 2008; Table S1). These include: the SoxXYZAB‐multienzyme complex (without SoxCD) for the conversion of thiosulphate and other reduced sulphur compounds to sulphate; sulphide:quinone oxido‐reductase type I and VI and flavocytochrome c:sulphide dehydrogenase for the conversion of sulphide to sulphane; reversible dissimilatory sulphite reductase (DsrAB) in conjunction with the DsrMKJOP complex for the conversion of sulphide and elemental sulphur to sulphite; as well as membrane‐bound sulphite dehydrogenase (SoeABC), reversible adenylylsulphate reductase (AprAB) and sulphate adenylyltransferase (Sat) for the conversion of sulphite to sulphate (Markert et al., 2007; Nakagawa & Takai, 2008; Frigaard & Dahl, 2009; Li et al., 2018; Reveillaud, Anderson, Reves‐Sohn, Cavanaugh, & Huber, 2018). Our genomic analyses further revealed the presence of a group 1e uptake [NiFe] hydrogenase (HyaAB) for hydrogen oxidation (Petersen et al., 2011; Thiel et al., 2012; Li et al., 2018; Reveillaud et al., 2018; Yang et al., 2019; but see Mitchell, Leonard, Delaney, Girguis, & Scott, 2019).

### 3.5 Carbon metabolism

Core enzymes for autotrophic carbon acquisition via the Calvin‐Benson‐Bassham cycle and the reductive tricarboxylic acid cycle were detected, such as RuBisCO form II (cbbM), phosphoribulokinase, ATP‐citrate lyase, 2‐oxoglutarate:ferredoxin oxidoreductase (KorABDG), and fumarate reductase (Table S1). We also discovered several genes for heterotrophic metabolism, including a NAD‐dependent formate dehydrogenase, and an anaerobic dimethyl sulphoxide (DMSO) reductase.

### 3.6 Nitrogen metabolism

The *L. barhami* symbiont genome encodes genes for both assimilatory and dissimilatory nitrate reduction (Table S1). Nitrate is reduced to nitrogen gas in a stepwise fashion involving the enzymes periplasmic nitrate reductase (Nap), nitrite reductase (Nir), nitric oxide reductase (Nor) and nitrous oxide reductase (Nos) (Li et al., 2018; Reveillaud et al., 2018; Yang et al., 2019). We further detected an octaheme tetrathionate reductase that was previously shown to reduce nitrite to ammonia in vent‐associated tubeworm symbionts (Robidart et al., 2011; Gardebrecht et al., 2012). In contrast to other genome reports (Li et al., 2018; Reveillaud et al., 2018), we found no evidence for the presence of a membrane‐bound respiratory nitrate reductase (Nar).

### 3.7 Host‐symbiont interactions

Our analyses reveal that the *L. barhami* symbiont genome contains a variety of genes for the biosynthesis of auxins and nodulation factors (Nod, Nol; Table S1 and S2), which play pivotal roles in bacterial invasion of host roots in *Rhizobia*‐legume symbioses (e.g., Dénarié & Cullimore, 1993; Gage, 2004; Buhian & Bensmihen, 2018). In addition, we found various genes coding for methyl‐accepting chemotaxis proteins, type IV pili, flagellar proteins, transporters and biosynthetic proteins for bacterial surface polysaccharides, general secretion pathway proteins, type I, II, IV and VI secretion systems, Colicin V, hemolysins, ankyrins, collagenases and pathogen‐related proteases (e.g., HtrA, DegP, Lon; Table S1 and S2), all of which have been suggested to be important for host invasion and cell permeabilization in diverse host‐microbe associations (Jones, Bolken, Jones, Zeller, & Hruby, 2001; Takaya et al., 2003; Hoy et al., 2012; Li et al., 2018; Reveillaud et al., 2018; Yang et al., 2019).

## 4 DISCUSSION

While several tubeworm symbiont genomes have been sequenced (Li et al., 2018; Yang et al., 2019), the present assemblies remain unfinished due to challenges inherent to metagenomic data sets, including strain‐level heterogeneity, interspecies repeat regions and low or uneven read coverage (e.g., Nurk, Meleshko, Korobeynikov, & Pevzner, 2017). We tried to remedy these challenges by using a combination of Illumina shotgun sequencing, ONT long‐read sequencing and chromosome conformation capture techniques to reconstruct the metagenome of the gammaproteobacterial endosymbiont of the cold seep tubeworm *L. barhami* from the Gulf of California. Our approach resulted in the first near chromosome‐level assembly for these symbionts. While this work will be a helpful guideline for other researchers working on metagenomic data, it provides an especially useful resource for further studies on the molecular adaptations, genetic variation and genome evolution of chemosynthetic bacteria.

Comparative genomic analyses indicated that the genome of the *L. barhami* symbiont is distinct from other tubeworm symbiont genomes in terms of both nucleotide sequence and structure. ANIs relative to other symbiont genomes were typically less than 93%, with the exception of the *P. echinospica* symbiont genome, which showed >97% identity. ANIs between the other seep‐associated symbionts (harboured by *L. luymesi*, *Escarpia spicata* and *Seepiophila jonesi*) as well as between the vent‐associated symbionts (harbored by *Riftia pachyptila*, *Ridgeia piscesae* and *Tevnia jerichonana*) were greater than 94%. Based on the currently recommended species‐level ANI cutoff of 94%–96% (Konstantinidis & Tiedje, 2005; Goris et al., 2007; Richter & Rosselló‐Móra, 2009; Meier‐Kolthoff, Auch, Klenk, & Göker, 2013), these findings suggest that the symbionts analysed in this study belong to at least three different bacterial species (and distinct bacterial strains), including the vent‐specific symbiont species *Ca. *Endoriftia persephone (confirming conclusions by Perez & Juniper, 2016) and two undescribed seep‐specific symbiont species. While our results imply a potential sister relationship between the symbionts of *L. barhami* and *P. echinospica*, future comparative genomic studies will need to reassess how representative these phylogenetic placements are once taxonomic sampling and genome information becomes available for more tubeworm symbiont species and strains.

Despite sequence similarities in homologous regions all symbiont genomes showed a marked amount of structural rearrangements, such as translocations, indels and inversions, as well as differences in the presence of several gene clusters. This level of variation contrasts markedly with analyses based on the *16S* rRNA gene, which have so far implied that tubeworm symbionts are genetically very similar, at least within vent and seep groups (McMullin et al., 2003). In contrast to prevailing beliefs, recent studies have shown that tubeworm symbionts can have varying degrees of intrahost diversity (Zimmermann et al., 2014; Reveillaud et al., 2018; Polzin, Arevalo, Nussbaumer, Polz, & Bright, 2019; Breusing, Franke, & Young, 2020) – a finding that is supported by our analyses. Despite potential inter‐strain competition, symbiont heterogeneity is predicted to be maintained if symbiont strains are functionally distinct and thereby promote adaptation of their hosts to fluctuating environmental conditions (Zimmermann et al., 2014; Perez & Juniper, 2016; Reveillaud et al., 2018; Polzin et al., 2019). The level of polymorphism detected in this study appears to be markedly higher than previously reported values (6.84 SNVs/kbp as opposed to 0.5–2.24 SNVs/kbp) (Reveillaud et al., 2018; Polzin et al., 2019), which could be related to differences in the diversity of free‐living symbiont populations at the time of host infection and/or differences in transmission modes and timing among symbiont and host species. The symbiont infection process has only been clearly documented in the *Riftia‐*Endorifta association, although the molecular mechanisms that establish and maintain symbioses in vestimentiferan tubeworms are still poorly understood (Nussbaumer et al., 2006; Klose et al., 2015). Nussbaumer et al. (2006) showed that *Riftia* tubeworms acquire their symbionts transdermally during the larval stage from a free‐living Endoriftia population that is present in the hydrothermal fluids. The symbionts subsequently initiate a profound metamorphosis of the mesoderm into trophosomal tissue. How symbionts are transmitted in seep‐associated host species is largely unknown, although the infection process probably differs from that of vent‐associated symbionts, considering the complex plant‐like anatomical and ecological adaptations of seep tubeworms to their sediment‐based environment (Cordes et al., 2005; Boetius, 2005). The *L. barhami* symbiont genome encodes a variety of nodulation factors, auxin biosynthesis proteins, chemoreceptors and outer membrane components, such as lipopolysaccharides, capsular polysaccharides, exopolysaccharides and β‐glucans. All of these compounds are essential for host‐symbiont encounter, bacterial adhesion and penetration of host cells in the *Rhizobia*‐legume symbiosis (e.g., Fraysse, Couderc, & Poinsot, 2003; Downie, 2010; Marczak, Mazur, Koper, Żebracki, & Skorupska, 2017). Although we currently lack histological evidence to support the following assumptions, it is possible that the host colonization mechanism in seep‐associated tubeworm symbionts is similar and involves invasion of preinfectious stages from the seep sediment through the tubeworm “root” system.

The *L. barhami* symbiont shows broad similarity in metabolic gene content to other tubeworm symbionts, having the potential to use both sulphide and hydrogen as energy sources for chemosynthesis as well as organic compounds for heterotrophic growth, including formate (as electron donor) and DMSO (as electron acceptor). Previous studies indicated that vestimentiferan symbionts are metabolically highly flexible and might alternate between different carbon assimilation strategies depending on the availability of electron donors and acceptors as well as micronutrients (Thiel et al., 2012; Li et al., 2018; Reveillaud et al., 2018; Yang et al., 2019). Such metabolic plasticity is predicted to provide a selective advantage under environmentally dynamic conditions (e.g., Anantharaman, Breier, Sheik, & Dick, 2013; Sanders, Beinart, Stewart, Delong, & Girguis, 2013), which are frequently encountered at hydrothermal vents (where fluid flows can change within minutes) (e.g., Johnson, Beehler, Sakamoto‐Arnold, & Childress, 1986; Johnson, Childress, Beehler, & Sakamoto, 1994), and cold seeps (where seepage flux can shift within a few days) (Tryon, Brown, & Torres, 2002). The importance of hydrogen as electron donor in vestimentiferan symbioses has recently been questioned given that respirometric measurements in *Riftia pachyptila* provided little evidence for coupling of hydrogen consumption to carbon incorporation (Mitchell et al., 2019). The authors suggested that hydrogen is potentially a significant energy source for vestimentiferan symbionts in their free‐living phase, but might be mostly used for redox‐homeostasis within the trophosome. Group 1e [NiFe] hydrogenases, which are encoded in the genomes of all tubeworm symbionts analysed so far, function bidirectionally and can evolve H_2_ through electron bifurcation (Greening et al., 2016), thereby allowing endergonic reactions under thermodynamically unfavourable conditions.

Our analyses imply that tubeworm symbionts encode a common set of core genes related to energy metabolism, cell signalling and translation as well as different, habitat‐specific gene clusters that are characteristic for either vent or seep‐associated symbiont groups and are primarily involved in signal transduction processes. Individual symbiont lineages, by contrast, appear to be more defined by genes that are important for antiviral defence. Bacteriophages are the main cause of bacterial mortality in the marine environment and are often highly abundant in cold‐seep sediments (Fuhrmann, 1999; Suttle, 2005; Kellogg, 2010). While symbionts might be protected from infections while residing inside the host cells, antiviral defence mechanisms are probably needed during their free‐living phase. The *L. barhami* symbiont contains a unique CRISPR/Cas3 system that comprises two CRISPR arrays not found in other tubeworm symbiont genomes. CRISPR‐Cas3 type I‐E involves incorporation of foreign DNA into a CRISPR array via the Cas1/Cas2 complex and subsequent transcription of the modified array sequence into a precurser CRISPR (cr) RNA (Hochstrasser & Doudna, 2015). The crRNA is cleaved by Cas6 into shorter fragments that are further processed for viral DNA interference by the Cas3‐Cascade complex containing Cas5e, Cas6, Cas7 (Cse4), Cas8 (Cse1) and Cas11 (Cse2) (Hochstrasser & Doudna, 2015; Rath, Amlinger, Rath, & Lundgren, 2015). Perez & Juniper (2016) suggested that variation in CRISPR array sequences between symbiont species and populations is probably an adaptation to geographic or habitat‐specific differences in viral strains. Although little is known about the composition of viral communities at deep‐sea cold seeps and vents, global virome studies have shown that viral assemblages differ significantly between oceanic regions (Angly et al., 2006), indicating that viruses might be important drivers of bacterial population structure in marine systems.

Many of the lineage‐specific genes in the *L. barhami* symbiont genome had no functional annotation. Although lineage‐specific (orphan) genes were long considered biologically irrelevant, there is increasing evidence that they promote the emergence of adaptive traits and novel biological functions that ultimately delineate different evolutionary entities (e.g., Wilson et al., 2005; Khalturin, Hemmrich, Fraune, Augustin, & Bosch, 2009; Tautz & Domazet‐Loso, 2011; Johnson, 2018). In bacteria, orphan genes have been shown to play significant roles in different metabolic pathways, cell wall biogenesis, biofilm formation, motility, virulence and pathogenicity, as well as interspecies competition (Wilson et al., 2005; Hu et al., 2009; Wang et al., 2011; Koskiniemi et al., 2012; Entwistle, Zueqiong, & Yanbin, 2019; Ross et al., 2019). Further analyses of orphan genes in the *L. barhami* symbiont might provide important insights into how this symbiont adapts to its specific ecological niche, how it discriminates among distinct host species and genotypes and how it can coexist with other closely related symbiont strains in the host trophosome.

In conclusion, this study reports a highly contiguous genome assembly for the chemosynthetic endosymbiont of the cold seep tubeworm *L. barhami* from the Gulf of California. Our analyses reveal significant overlap in metabolic capacities and antiviral defence systems between the *L. barhami* symbiont and other tubeworm symbionts and provide insights into potential mechanisms of symbiont transmission in seep‐associated tubeworm hosts, implying that the symbiont infection process might involve signalling molecules that are also important in plant‐associated rhizobia. The *L. barhami* symbiont further contains a variety of lineage‐specific features, including a CRISPR/Cas3 system, possibly reflecting adaptations to viral pathogens and other habitat characteristics at the Gulf of California seeps. These results contribute to continued efforts to provide high‐quality reference genomes of chemosynthetic tubeworm symbionts so that we can better understand their metabolism, diversity and evolutionary ecology.

## AUTHOR CONTRIBUTIONS

C.B., and D.T.S. designed the study with advice from R.C.Y., conducted the molecular laboratory work with help from S.S. and performed the bioinformatic analyses. C.B. wrote the paper. D.T.S, S.S., A.Z.W., and R.C.Y. edited the manuscript.

## Supporting information

Supplementary Material

## Data Availability

The final genome assembly including associated raw reads from Nanopore and Illumina sequencing are available in GenBank under BioProject number PRJNA609990. Genome annotations can be found as GFF and TSV files in the Supporting Information. Host mitochondrial *COI* sequences have been deposited in GenBank under accession numbers MT145916–MT145921 and MK047330.
