# Supplementary material for: High‐contiguity genome assembly of the chemosynthetic gammaproteobacterial endosymbiont of the cold seep tubeworm Lamellibrachia barhami
Source: Mol Ecol Resour. 2020 Jul 24;20(5):1432–44. doi: 10.1111/1755-0998.13220 (PMC7540712; doi:10.1111/1755-0998.13220)
Supplement: Supplementary file 1 — Supplementary Material [file MEN-20-1432-s001.zip › men13220-sup-0009-Methods.docx]

**Supplementary Methods for: High-contiguity genome assembly of the chemosynthetic gammaproteobacterial endosymbiont of the cold seep tubeworm *Lamellibrachia barhami***

Corinna Breusing, Darrin T. Schultz, Sebastian Sudek, Alexandra Z. Worden, C. Robert Young

**Supplementary Method 1:** CHAOS DNA extraction

**Step 1:** Tissue lysis

1. Prepare buffers and solutions. Mix the CHAOS solution for about an hour.
2. Place a small amount of tissue into a 2 ml tube and add 2 ml of CHAOS solution.
3. Leave for five days at room temperature before continuing with the extraction.

**100 ml CHAOS solution:**

4 M Guanidine thiocyanate 50 g

0.5% N-lauroyl sarcosine sodium 0.5 g

25 mM Tris pH 8 2.5 ml

0.1 M 2-mercaptoethanol 0.7 ml

ddH_2_O up to 100 ml

**100 ml Phenol extraction buffer (PEB):**

1 M Tris + 0.1 M EDTA 10 ml

10% SDS 1 ml

ddH_2_O up to 100 ml

**Additional chemicals:**

7.5 M ammonium acetate

Phenol

Chloroform

RNAse A

100% ethanol

70% ethanol

5 mg/ml glycogen

**Step 2:** Phenol:chloroform extraction

1. Use 2 ml tubes. Make sure that tissues have been sufficiently lysed. Add 400 µl tissue solution and 400 µl PEB, invert 10x and incubate for at least 30 min. Add 800 µl 1:1 phenol:chloroform mixture, invert 10x and incubate for 10 min.
2. Centrifuge at 8000 rcf for 10 min.
3. Move 400 µl aqueous layer to a new 2 ml tube. Add 10 µl RNAse A. Invert 10x and incubate at room temperature for 5 min.
4. Add 800 µl 1:1 phenol:chloroform mixture, invert 10x and incubate for 10 min.
5. Centrifuge at 8000 rcf for 10 min.
6. Move 400 µl aqueous layer to a new 1.5 ml tube.
7. Add 160 µl ammonium acetate, 2 µl glycogen and 800 µl 100% ethanol. Invert gently and incubate DNA at -20 °C for 1 h.
8. Centrifuge at 12000 rcf for 20 min.
9. Discard the supernatant and wash with 600 µl 70% ethanol.
10. Centrifuge at 12000 rcf for 10 min.
11. Repeat steps 9 and 10.
12. Remove all ethanol remains and air-dry the pellet under the fume hood.
13. Add 100 µl 10 mM Tris buffer pH 8 and incubate to dissolve DNA.

**Supplementary Method 2:** CsCl Bisbenzimide Gradient Separation

**Day 1:** Ultracentrifugation

1. Quantify amount of DNA with Qubit. Aim for ≥20 ug as starting material.
2. Prepare CsCl solution (1.1 g/ml). Add 110 g CsCl to 100 ml MilliQ and stir until dissolved. Store at room temperature.
3. Prepare bisbenzimide solution (1.1 mg/ml). Add 11 mg bisbenzimide to 10 ml MilliQ, vortex thoroughly and wrap in aluminum foil. Store at 4 °C.
4. Prepare DNA mixture. For 20 µg DNA mix 5 ml CsCl solution, 10 µl bisbenzimide solution and the respective amount of DNA. The proportion of DNA:bisbenzimide should be 2:1 (e.g., 20 µg DNA + 10 µg bisbenzimide).
5. Fill CsCl solution into balance ultracentrifuge tube. Use a P1000 up to about 5 ml, then top off with P200 long (gel-loading) tips. To avoid spilling insert solution rapidly at the base of the tube neck. To remove bubbles tap tubes gently on the bench.
6. Fill DNA solution into another ultracentrifuge tube. Top off with CsCl solution.
7. Weigh tubes and make sure they are less than 10 mg different.
8. Seal the tubes using the heat sealer. Verify each tube is leak-free by squeezing forcefully by hand.
9. Double check the tube pairs remain balanced.
10. Place tube into VTi90 rotor with balanced pairs opposite each other. Place spacer on top of tube in well.
11. Seal rotor well with plug and gasket using torque wrench to 120 inch/lbs.
12. Use the following settings on ultracentrifuge: 48:00 hours, 158,000 rcf, 20 °C, coast deceleration, VTi90 rotor.

**Day 2:** Fractionation

1. After spin, retrieve tubes taking care to avoid disturbing the gradients. Place rotor on base to open, and have second person hold onto rotor tightly. Remove spacer using removal tool/large forceps. Be careful, tube may stick to spacer. Process gradients as quickly as possible to minimize diffusion.
2. Fix the tube to a ring stand clamp. Wipe the top and base with 70% ethanol. Illuminate under UV light and determine number of fractions.
3. Pre-fill 10 ml syringe and tubing with MilliQ water. Fill syringe to the top. Push depressor until water exits needle, ensure no visible bubbles in syringe or tubing.
4. Set-up on pump and run once until water exits needle.
5. Carefully pierce the top of the tube in a smooth and controlled manner with the 23-gauge 1” needle attached to the syringe pump tubing. Carefully pierce the bottom of the tube in a smooth and controlled manner with a 23-gauge 1” needle, then discard needle. Using a syringe pump, inject MilliQ water into the top of the tube through the 23-gauge 1” needle and collect drops from below in 2.0 ml microcentrifuge tubes.
6. Using a 10 ml BD Scientific plastic syringe, run syringe pump at a rate of 0.5 ml/minute. For 6 fractions stop after every 850 µl.
7. Add 1 ml butanol to each fraction. Invert 10 times. Spin at 12,000 rcf for 1 min. Remove upper butanol phase and discard.
8. Repeat previous step 3 times.
9. On third time take lower aqueous phase with P1000 and transfer into 2 ml tubes or directly onto Amicon columns (100 k).

Day 3: Cleanup of DNA

1. Load columns with DNA-CsCl solution.
2. Spin at 3,222 rcf for 10 min.
3. Add 1 ml TE pH 8 buffer. Spin at 3,222 rcf for 10 min. Discard filtrate.
4. Repeat previous step 3 more times.
5. Pipet DNA solution out of the column using P200 long tips. Transfer to 1.7 ml tubes.
6. Quantify the amount of DNA in each fraction with Qubit.
